# National Child Passenger Safety Week — September 15–21, 2013

**Published:** 2013-09-13

In the United States, motor vehicle–related injuries are a leading cause of death among children (1). In 2011, a total of 656 passenger vehicle occupants aged 0–12 years died as a result of a crash (2). During 1975–2011, child restraints saved an estimated 9,874 lives of children aged 0–4 years (2). Seating position also contributes to child passenger safety. To keep child passengers as safe as possible, drivers should properly restrain children aged <13 years in a back seat and follow the American Academy of Pediatrics’ child passenger safety recommendations (3).

For 2013, National Child Passenger Safety Week is September 15–21. As part of the campaign, September 21 is designated as National Seat Check Saturday, when drivers with child passengers are encouraged to visit a child safety seat inspection station to have a certified technician inspect their car seat and give hands-on advice free of charge. Additional information and an inspection station locator are available from the National Highway Traffic Safety Administration at http://www.nhtsa.gov/Safety/CPS. Promotional materials (in English and Spanish) are available at http://www.trafficsafetymarketing.gov/cps.
